# Quantitative Evaluation of Human Cerebellum-Dependent Motor Learning through Prism Adaptation of Hand-Reaching Movement

**DOI:** 10.1371/journal.pone.0119376

**Published:** 2015-03-18

**Authors:** Yuji Hashimoto, Takeru Honda, Ken Matsumura, Makoto Nakao, Kazumasa Soga, Kazuhiko Katano, Takanori Yokota, Hidehiro Mizusawa, Soichi Nagao, Kinya Ishikawa

**Affiliations:** 1 Department of Neurology and Neurological Science, Graduate School, Tokyo Medical and Dental University, Bunkyo, Tokyo, Japan; 2 Laboratory for Motor Learning Control, RIKEN Brain Science Institute, Wako, Saitama, Japan; 3 Japan Society for the Promotion of Science Research Fellow, Chiyoda, Tokyo, Japan; 4 KATANO TOOL SOFTWARE, Ichikawa, Chiba, Japan; 5 Nozomi Higher Brain Function Laboratory, Nozomi Hospital, Ina-machi, Kita-adachi-gun, Saitama, Japan; University Zurich, SWITZERLAND

## Abstract

The cerebellum plays important roles in motor coordination and learning. However, motor learning has not been quantitatively evaluated clinically. It thus remains unclear how motor learning is influenced by cerebellar diseases or aging, and is related with incoordination. Here, we present a new application for testing human cerebellum-dependent motor learning using prism adaptation. In our paradigm, the participant wearing prism-equipped goggles touches their index finger to the target presented on a touchscreen in every trial. The whole test consisted of three consecutive sessions: (1) 50 trials with normal vision (BASELINE), (2) 100 trials wearing the prism that shifts the visual field 25° rightward (PRISM), and (3) 50 trials without the prism (REMOVAL). In healthy subjects, the prism-induced finger-touch error, i.e., the distance between touch and target positions, was decreased gradually by motor learning through repetition of trials. We found that such motor learning could be quantified using the “adaptability index (*AI*)”, which was calculated by multiplying each probability of [acquisition in the last 10 trials of PRISM], [retention in the initial five trials of REMOVAL], and [extinction in the last 10 trials of REMOVAL]. The *AI* of cerebellar patients less than 70 years old (mean, 0.227; n = 62) was lower than that of age-matched healthy subjects (0.867, n = 21; p < 0.0001). While *AI* did not correlate with the magnitude of dysmetria in ataxic patients, it declined in parallel with disease progression, suggesting a close correlation between the impaired cerebellar motor leaning and the dysmetria. Furthermore, *AI* decreased with aging in the healthy subjects over 70 years old compared with that in the healthy subjects less than 70 years old. We suggest that our paradigm of prism adaptation may allow us to quantitatively assess cerebellar motor learning in both normal and diseased conditions.

## Introduction

The cerebellum plays an important role in motor control [1]. Experimental studies using adaptation of ocular reflexes and eyeblink conditioning have consistently suggested that the cerebellum controls gain and timing of movements through learning [1–3]. Patients with cerebellar diseases exhibit signs of ataxia that include imbalance and incoordination [4, 5], as well as impaired motor learning, which has been revealed with the paradigms of eyeblink conditioning [6–8], adaptation of ocular reflexes [9, 10], and adaptation of forelimb movements [11–15]. However, these paradigms of motor learning have been used very rarely for the diagnosis and treatment of cerebellar diseases clinically due to technical or practical reasons. Additionally, the accuracy of motor learning assessment using these paradigms largely depends on the competence of each subject, or the time or number of trials needed for evaluation. Thus, it is still unknown how far motor learning and cerebellar ataxia with signs of such as incoordination or equilibrium disturbance correlate with each other.

In this study, we developed a new application of human prism adaptation by referring to the studies of Thach’s group [13, 16, 17]. In our paradigm, simple hand-reaching movement is used instead of dart throwing, with the minimum number of cheap instruments that require a relatively small space. By using our paradigm, we were able to quantify motor learning within 30 min. We examined how motor learning capacity is degraded by cerebellar diseases or aging for more than 100 patients with cerebellar ataxia and healthy subjects. We propose that the adaptability index (*AI*), calculated on the basis of the data obtained using our paradigm, as a sensitive marker of human cerebellar motor learning for the practical diagnosis of cerebellar diseases.

## Materials and Methods

The experimental procedure was approved by the Ethics Committee of Tokyo Medical and Dental University.

### Participants

After obtaining written informed consent, 38 healthy subjects (S1 Table) and 77 patients with degenerative cerebellar diseases (Table 1) were studied. We defined healthy subjects as those without any obvious neurological disorders and any disturbances in daily living. To determine aging effects on motor learning, we divided healthy subjects into two groups: 21 non-elderly healthy subjects less than 70 years old (HN; mean age, 49.0; range, 28–68) and 17 elderly ones ≥ 70 years old (HE; mean age, 78.4; range, 72–88). Likewise, the 77 patients with cerebellar diseases were divided at 70 years old: 62 non-elderly subjects (CN; mean age, 54.7; range, 29–69) and 15 elderly ones (CE; mean age, 74.8; range, 70–83). Among the 77 patients with cerebellar diseases, 44 had spinocerebellar ataxia (SCA) as confirmed by genetic testing [18–20], and 11 had sporadic cortical cerebellar atrophy (CCA) [21, 22]. The remaining 22 patients had multiple system atrophy (MSA) [23], in which three MSA-P and 15 MSA-C patients had clear signs of cerebellar ataxia while the remaining four MSA patients lacked them. Here, we defined these four patients as “pure parkinsonian MSA patients” (Table 1).

**Table 1:** Characteristics of patients with cerebellar diseases.

Non-elderly cerebellar ataxia patients (CN)													
ID	Age (year) / Gender / Handedness	Diagnosis	Disease duration (Month)	SARA	9HPT (sec)	*AI*	ID	Age (year) / Gender / Handedness	Diagnosis	Disease duration (Month)	SARA	9HPT (sec)	*AI*
CN1	39 / M / R	SCA6	114	10	26.65	0.360	CN32	45 / M / R	MJD	85	6	28.13	0.420
CN2	62 / F / R	SCA6	147	14	30.25	0.320	CN33	46 / M / L	MJD	163	7	29.85	0.640
CN3	62 / M / R	SCA6	142	14	39.16	0.000	CN34	46 / F / R	MJD	36	9	24.28	0.480
CN4	63 / M / R	SCA6	153	26	140.82	0.012	CN35	48 / M / R	MJD	90	14.5	40.1	0.126
CN5	66 / F / R	SCA6	96	21.5	51.91	0.080	CN36	48 / M / L	MJD	159	24	72.44	0.336
CN6	66 / M / R	SCA6	100	11	39.34	0.000	CN37	60 / M / R	MJD	174	13	35.75	0.000
CN7	68 / F / R	SCA6	36	2.5	23.78	0.400	CN38	56 / M / R	SCA2	84	6.5	27	0.100
CN8	53 / M / R	SCA31	235	6.5	23.88	0.120	CN39	36 / F / R	SCA8	147	10	37.03	0.192
CN9	56 / F / R	SCA31	86	5.5	23.78	0.324	CN40	41 / F / R	SCA8	154	13.5	35.9	0.032
CN10	63 / F / R	SCA31	44	7	33.16	0.000	CN41	69 / M / R	SCA36	184	12	42.84	0.056
CN11	64 / F / R	SCA31	99	19	44.78	0.000	CN42	69 / F / R	DRPLA	91	14.5	36.16	0.270
CN12	66 / M / R	SCA31	99	10.5	29.53	0.320	CN43	48 / M / R	MSA-C	68	14.5	47.03	0.240
CN13	66 / F / R	SCA31	182	13.5	46.88	0.000	CN44	56 / M / R	MSA-C	40	15	43	0.168
CN14	68 / M / R	SCA31	72	15.5	50.53	0.054	CN45	57 / M / R	MSA-C	51	6.5	24.07	0.384
CN15	68 / M / L	SCA31	104	11	23.78	0.144	CN46	58 / M / R	MSA-C	19	12	46.22	0.144
CN16	29 / F / R	CCA	198	5	33.81	0.000	CN47	58 / M / R	MSA-C	28	13.5	26.28	0.504
CN17	39 / M / R	CCA	100	9	29.03	0.420	CN48	59 / F / R	MSA-C	20	8	26.54	0.120
CN18	42 / F / R	CCA	204	10	49.5	0.080	CN49	61 / M / R	MSA-C	36	12.5	32.41	0.300
CN19	45 / F / R	CCA	27	4.5	24.91	0.280	CN50	62 / M / R	MSA-C	76	11.5	39.68	0.096
CN20	45 / M / R	CCA	294	10.5	57.84	0.064	CN51	63 / F / R	MSA-C	25	10.5	34.81	0.064
CN21	48 / F / R	CCA	115	6.5	20.87	0.280	CN52	64 / M / R	MSA-C	15	11.5	32.57	0.096
CN22	59 / F / R	CCA	349	13.5	34.94	0.000	CN53	64 / M / R	MSA-C	36	8	24.15	0.324
CN23	63 / F / R	CCA	281	16	40.75	0.120	CN54	64 / F / R	MSA-C	38	10	49	0.000
CN24	65 / M / R	CCA	206	10.5	29.09	0.108	CN55	65 / F / R	MSA-C	40	12.5	40.56	0.016
CN25	31 / F / R	MJD	51	7.5	29.34	0.360	CN56	56 / M / R	MSA-P	27	7	37.31	0.486
CN26	33 / M / R	MJD	75	11.5	23.78	0.480	CN57	56 / M / R	MSA-P	45	12.5	27.91	0.378
CN27	36 / F / R	MJD	72	16	28.44	0.324	CN58	60 / M / R	MSA-P	33	8	27.47	0.140
CN28	37 / F / R	MJD	14	5.5	25.94	0.120	CN59	56 / M / R	PP-MSA	42	1.5	26.91	0.800
CN29	41 / F / R	MJD	269	14.5	55.91	0.168	CN60	63 / M / R	PP-MSA	16	10	25.5	0.640
CN30	43 / F / R	MJD	124	12.5	27.25	0.252	CN61	65 / M / R	PP-MSA	20	7.5	32.09	0.540
CN31	44 / M / R	MJD	166	9	29.22	0.196	CN62	65 / M / R	PP-MSA	30	10	33.21	0.576

Elderly cerebellar ataxia patients (CN)													
ID	Age (year) / Gender / Handedness	Diagnosis	Disease duration (Month)	SARA	9HPT (sec)	*AI*	ID	Age (year) / Gender / Handedness	Diagnosis	Disease duration (Month)	SARA	9HPT (sec)	*AI*
CE1	70 / M / R	SCA6	79	11	26.41	0.432	CE9	76 / F / R	SCA31	252	23	83.1	0.072
CE2	71 / M / R	SCA6	36	16	37.41	0.042	CE10	79 / F / R	SCA31	64	12	33.93	0.270
CE3	71 / M / R	SCA6	225	15	54.53	0.000	CE11	79 / F / R	SCA31	237	17	37.1	0.256
CE4	74 / M / R	SCA6	210	19.5	56.28	0.060	CE12	77 / M / R	CCA	84	7	27.37	0.012
CE5	72 / F / R	SCA31	49	7	27.03	0.000	CE13	83 / M / R	CCA	84	18	39.9	0.008
CE6	72 / F / R	SCA31	276	15.5	35.22	0.108	CE14	72 / M / L	MSA-C	37	14.5	50.57	0.256
CE7	74 / F / R	SCA31	48	13.5	35.9	0.270	CE15	77 / F / R	MSA-C	71	10.5	34.28	0.324
CE8	75 / M / R	SCA31	247	17.5	47.53	0.000							

CN = non-elderly (< 70 years old) patients. CE = elderly (≥ 70 years old) patients. SCA2, 6, 8, 31, 36 = spinocerebellar ataxia type 2, 6, 8, 31, 36. MJD = Machado-Joseph disease. DRPLA = dentatorubral-pallidoluysian atrophy. CCA = cortical cerebellar atrophy. MSA-C = multiple system atrophy with predominant cerebellar ataxia. MSA-P = multiple system atrophy with predominant parkinsonism. PP-MSA = pure parkinsonian MSA without cerebellar ataxia. M = male. F = female. R = right. L = left. SARA = Scale for the assessment and rating of ataxia. 9HPT = nine-hole peg test. *AI* = adaptability index.

The CN group was further classified into the diseases predominantly affecting the cerebellar cortex (CBL group) [SCA6, SCA31, and CCA; n = 24], and the ones that were accompanied by extra-cerebellar degenerations (CBL+ group) [SCA2, Machado-Joseph disease (MJD), SCA8, SCA36, DRPLA, MSA-C and MSA-P; n = 34] [24–29]. The ataxia of all the patients was rated using the Scale for the Assessment and Rating of Ataxia (SARA) [30], and by the 9-Hole Peg Test (9HPT) using a Rolyan 9-hole peg test apparatus and plastic one-piece model. Disease onset age was defined as the age when the patients first noticed the signs of cerebellar ataxia. All the participants were naive to the experiments for their first test, except for five MSA patients who were tested every 3 to 6 months to track their disease progressions, and their visual acuity was normal or corrected by spectacles.

### Experimental apparatus

**Fig 1:**
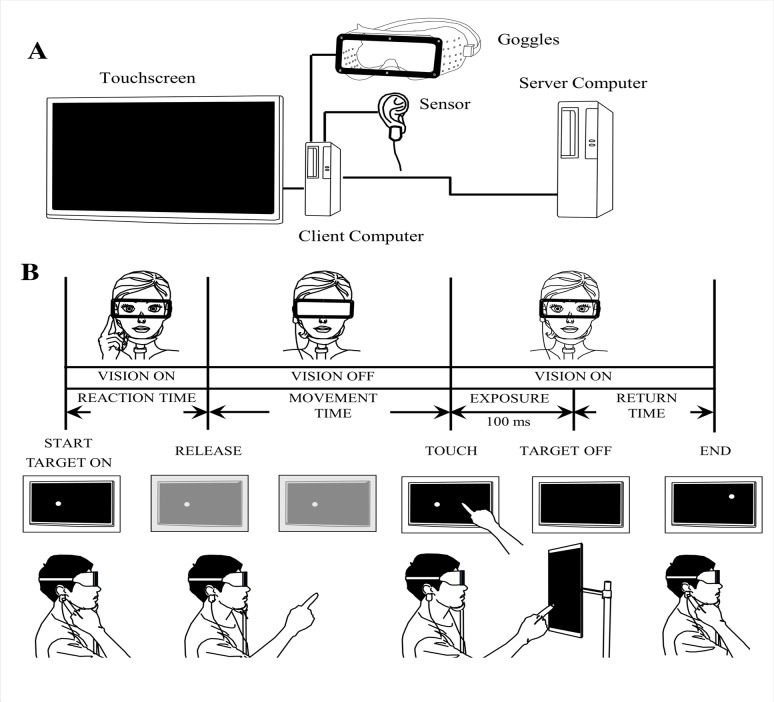
Scheme for prism adaptation of hand-reaching. (A) The experimental apparatus consists of a sensor on the participant’s right earlobe, goggles equipped with an electrically controlled shutter with a plastic or Fresnel prism plate, a touchscreen, and two computers. (B) Time sequence of a single trial shown from left to right. Every trial starts from the touch of a participant’s index finger at the sensor on the right earlobe. As soon as the participant releases their index finger from the sensor, vision is blocked by the shutter (MOVEMENT TIME). Immediately after reaching the touchscreen (TOUCH), the goggles become transparent, and the participant recognizes how their index finger deviated/hit the target for 100 ms (EXPOSURE). Subsequently, the target disappears (TARGET OFF) and the participant returns their index finger to the original position in preparation for the next trial.

The apparatus consisted of two Windows 7 personal computers (HP Compaq 8200 Elite, CT, USA), one server for task control and one client for data sampling and analysis, a 23-inch touchscreen (HP 2310t, Hewlett-Packard Japan, Tokyo, Japan), custom-made goggles, and a sensor on the participant’s right earlobe (Fig. 1A). The touchscreen display resolution was 1920 (w) × 1080 (h) pixels (95.78 dpi; dot pitch, 0.265 mm). The goggles contained either a transparent plastic plate (16 × 4.3 cm^2^) or a Fresnel prism plate of the same size (LP25, Nihon Tokushu Kogaku Jushi, Tokyo, Japan), which shifted the visual field 25° rightward. The goggles were also fitted with an electrically controlled shutter (NSG UMU PRODUCTS, Chiba, Japan), opened by the command voltage pulse-on (100 V) and closed 10 ms after pulse-off. Software (Visual Reaching Task software, KATANO TOOL SOFTWARE, Chiba, Japan) based on LabVIEW2011 (National Instruments Japan, Tokyo, Japan) was used for controlling the touchscreen and shutter and for sampling and analyzing data.

### Hand-reaching task

The participants sat on a chair wearing the goggles, with their head loosely restrained by a chin rest. The touchscreen was set 320–530 mm in front of them depending on the length of their individual reach. Until a start signal was given, the participants touched the earlobe sensor with their right index finger (Fig. 1B). While touching the sensor, a target (white circle, 8 mm in diameter) appeared randomly at one of the 3 × 3 grid cells (width and height of each grid, 10 and 6.7 cm, respectively) except at the center of the touchscreen. Then, the participants were requested to reach their index finger to the target. Immediately after the participants released their index finger from the sensor, the electrically controlled shutter was changed from transparent to translucent by the computer, and their vision was blocked until 10 ms after the index finger touched the screen (Fig. 1B). This was intended to prevent visual online correction through detecting the error by viewing the trajectory of finger, just to mimic the dart throwing experiments in which no corrections could occur once after the dart was thrown. Subsequently, the shutter was reopened, which allowed the participants to see their finger and the target for 100 ms through the goggles. Then, the target on the touchscreen disappeared with a beep sound of short duration, and the participants were requested to return their index finger from the touchscreen to the sensor. After an interval of more than 200 ms, the next trial was started. When the participant failed to touch a touchscreen within 5 s after releasing their index finger from the sensor, such a trial was counted as a failure. Failure trial was not counted as a trial, and skipped to a new trial.

### Prism adaptation task

Before testing prism adaptation, all the participants familiarized themselves with the experimental setup by performing 100 trials without the prism. The test consisted of three consecutive sessions: (1) 50 trials with normal vision wearing the transparent plastic plate (BASELINE), (2) 100 trials wearing the prism, shifting the visual field 25° rightward (PRISM), and (3) 50 trials wearing the transparent plastic plate without the prism (REMOVAL). A short break of 0.5 min was made between sessions, during which examiners replaced the transparent plastic plate with the prism plate, or vice versa. The test was undertaken in a quiet dark room, and it took 20–30 minutes for each participant to complete the entire test.

### Data analysis

The finger-touch error, i.e., the distance between the touch position and the target on the touchscreen, was automatically measured and stored in the client computer for each trial. As the prism shifted the visual field horizontally, only the deviation in the horizontal plane was analyzed. The mean and standard deviation (SD) of the finger-touch error were calculated in each trial for each group of participants. The change in the variability of the finger-touch error during PRISM was evaluated for each participant as *e*/*d*, where *d* is the SD of the finger-touch error for the initial 20 trials of PRISM, and *e* is that for the last 20 trials of PRISM. The correct touch was determined when the finger-touch error was ≤ 25 mm, by referring to the mean + twice the SD of the finger-touch error of 21 HN subjects in the last 10 trials of BASELINE.

Three probabilities were calculated to evaluate adaptation: (1) the acquisition of adaptation (“*a*”) defined as the probability of correct touches in the last 10 trials of PRISM; (2) the retention of adaptation (“*b*”) defined as the probability of incorrect touches in the initial five trials of REMOVAL, and (3) the extinction of adaptation (“*c*”) designated as the probability of correct touches in the last 10 trials of REMOVAL. It should be noted that “*a*” and “*c*” are for correct touches, whereas “*b*” is for incorrect touch as the number of incorrect touch reflect retention of adaptation. Healthy individuals usually show high scores in *a*, *b*, and *c*. The adaptability index (*AI*) was calculated as *AI* = *a* × *b* × *c*. In addition, the time constant (*τ*) in the initial part of PRISM was also analyzed by referring to Martin *et al*. [13]. The adaptation curve in PRISM was drawn using the GraphPad Prism software (ver. 6.02, GraphPad Software, SDG, USA) and fitted as y = *α* · exp (-*t*/*τ*) + *β*, where *y* is the finger-touch error, *β* is the final value that the exponential decay function approaches, *α* is the distance from the finger-touch error at the initial trial of PRISM to the plateau *β*, *t* is the number of trials; and *τ* is the number of trials when the finger-touch error approached the (1- exp (-1)) = 63.2% of *α*. We then assessed which of the five parameters (*a*, *b*, *c*, *AI* and *τ*) best reflects cerebellar function using the cumulative frequency distribution receiver operating characteristic (ROC) curves by referring to Swets [31]. The sensitivity and specificity of these five parameters were determined by referring to Lalkhen and McCluskey [32]. We calculated the area under the ROC curve (AUC) to quantify the overall ability of the parameters to discriminate between patients with cerebellar disease and healthy subjects.

### Statistical analysis

The Mann-Whitney U-test or Kruskal-Wallis test for multiple comparisons was used to assess the differences in *AI* between healthy subjects and patients with cerebellar diseases, and among HN, HE, CN and CE groups. Post hoc comparisons using the Steel-Dwass test following the Kruskal-Wallis test were performed to determine which groups differed from each other. Cumulative frequency distributions of HN, HE, CN and CE groups were compared using the Kolmogorov-Smirnov test [33]. The distribution of *AI* in HE and HN groups were compared by the Ansari-Bradley test [34]. The AUCs of the five parameters were compared by DeLong's test [35]. Spearman’s rank order correlation was used to examine the relationship between the *AI* for individual patients and the SD of finger-touch error, between *AI* and SARA score, between *AI* and 9HPT, and between *AI* and disease durations. Spearman’s rank order correlation coefficients (*r*_s_) were calculated to determine the strength of the association between two variables. *p* < 0.05 was regarded as statistically significant. For these statistical analyses, GraphPad Prism software, Matlab software (Matlab2013a, MathWorks, MA, USA) and R software (version 3.1.0, The R Foundation for Statistical Computing, Vienna, Austria) were used. Unless otherwise stated, data were described as mean ± standard error of the mean (SEM).

## Results

### Prism adaptation of hand-reaching movement

**Fig 2:**
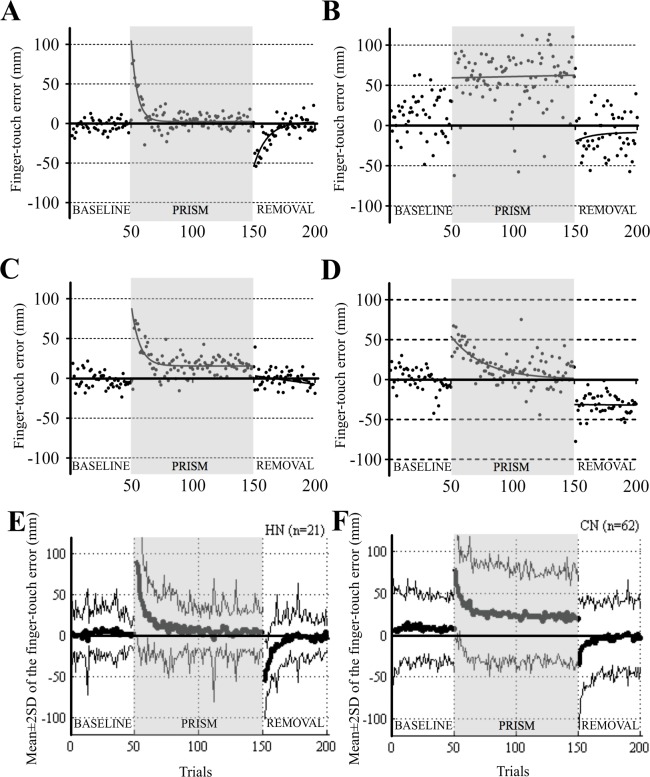
Adaptation curves in different subjects and in healthy and patient groups. (A)–(D) Adaptation curves in HN13 (A), CN4 (B), CN3 (C), and CN15 (D). The ordinate shows the finger-touch error represented by the distance (mm) from the target to the touch point. Positive values indicate rightward shifts and negative values indicate leftward shifts. The abscissa shows the trial numbers. Best-fitted exponential curves (for details, see Materials and Methods) are overlaid on the raw data. Whereas a normal subject (HN13) shows typical adaptation (A), patients with cerebellar diseases show three different patterns of impaired adaptation (B)–(D). (E) and (F) Average adaptation curves for 21 HN subjects (E) and 62 CN patients (F). Thick and thin curves show mean and mean ± 2SD, respectively.

Prism adaptation occurred quickly in healthy subjects, as shown in Fig. 2A [51-year-old healthy subject (HN13, S1 Table)]. Before wearing the prism (BASELINE), she touched the target correctly in most of the trials. At the initial few trials of PRISM, she touched rightward as the prism shifted her visual field rightward. After 30–40 trials, she was able to touch the target correctly following the acquisition of prim adaptation. When the prism was removed (REMOVAL), she touched leftward from the target owing to the retention of adaptation. By the time she finished 20 subsequent trials with normal vision, she was able to touch the target correctly due to the extinction of adaptation.

On the other hand, adaptation was impaired in patients with cerebellar diseases. A patient with SCA6 (CN4, 63 years old, SARA = 26; Table 1), irregularly missed the targets in BASELINE (Fig. 2B). He touched consistently the targets with a large rightward deviation showing no acquisition of adaptation in PRISM, and missed the targets similarly in REMOVAL as in BASELINE. However, not all patients showed such typical alterations. In some patients, the retention of adaptation was absent, which was noticed by the lack of leftward deviation at the initial trials of REMOVAL (CN3, 62 years old, SARA = 14; Fig. 2C), or the acquisition of adaptation was slow and extinction in REMOVAL was absent in a patient with SCA31 (CN15, 68 years old, SARA = 11; Fig. 2D).

Figs. 2E and 2F respectively show plots of the mean ± 2SD of finger-touch error for each trial in the HN and CN groups. In PRISM, the HN group quickly adapted, whereas the CN group very slowly and incompletely adapted, indicating the impaired acquisition of adaptation. In REMOVAL, the retention of adaptation was large in the HN group, but small in the CN group, showing little retention in the CN group. These results consistently suggest that the prism adaptation was impaired markedly in the CN group.

**Fig 3:**
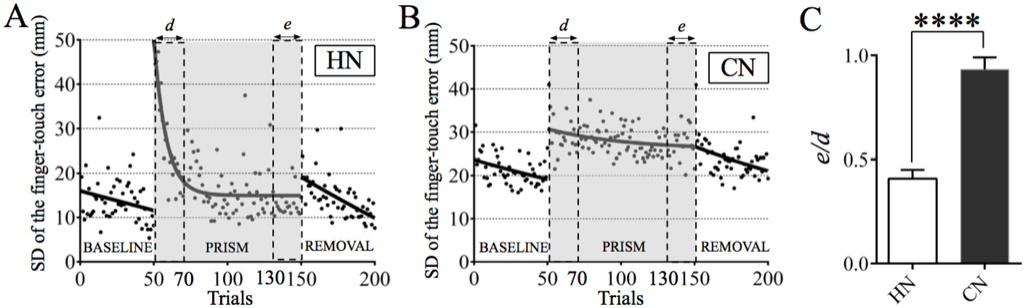
Variability of the finger-touch error in HN and CN groups. (A) Variability of the finger-touch error in 21 HN subjects. Each dot shows SD of the finger-touch error for every trial throughout BASELINE, PRISM, and REMOVAL. An interpolated curve was drawn by fitting with nonlinear regression. (B) Same analysis as (A) in patients with cerebellar diseases below 70 years old (CN). (C) Comparison of intertrial variability of the finger-touch error shown in A and B between the initial 20 and the last 20 trials. The ordinate shows the ratio (*e*/*d*) of the mean SD for the last 20 trials (*e*) in PRISM to that for the initial 20 trials (*d*) of PRISM. Note that the intertrial variability markedly decreased following adaptation in the HN group, but not in the CN group. **** *p* < 0.0001 by Mann-Whitney U-test. Error bar represents SEM.

We analyzed the variability of the finger-touch error in every trial by measuring its SD, and compared the variability between the HN and CN groups (Figs. 3A and 3B). In BASELINE of the HN group, the variability of the finger-touch error was distributed at 11.9–15.9 mm, and uniformly decreased as the trial number increased. At the start of PRISM, the variability of the finger-touch error increased owing to a shift of the visual field, but soon decreased to the level equivalent to the plateau of BASELINE. In contrast, the variability of the finger-touch error in BASELINE of the CN group (18.6–23.4 mm) was larger than that of the HN group. Nevertheless, the SDs tended to decrease gradually in both BASELINE and PRISM. When such a decrease in the variability of the finger-touch error during PRISM was compared between the CN and HN groups, it markedly decreased following the acquisition of adaptation in the HN group (*e*/*d* = 0.42 ± 0.04), but not in the CN group (*e*/*d* = 0.91 ± 0.05, *p* < 0.0001, Mann-Whitney U-test; Fig. 3C). Thus, the adaptation was impaired not only in the magnitude of the finger-touch error but also in its variability in the CN patients.

### Adaptability index (*AI*) as a quantitative marker for motor learning

We determined a quantitative marker that reflects motor learning for every participant tested. Because the mean + 2SD of the finger-touch error for the last 10 trials was 20.5 mm in BASELINE of the HN group, we set the normal deviation of the finger-touch error at this value. Then, we defined a trial as correct if the finger-touch error was within ± 25 mm for all the participants.

**Fig 4:**
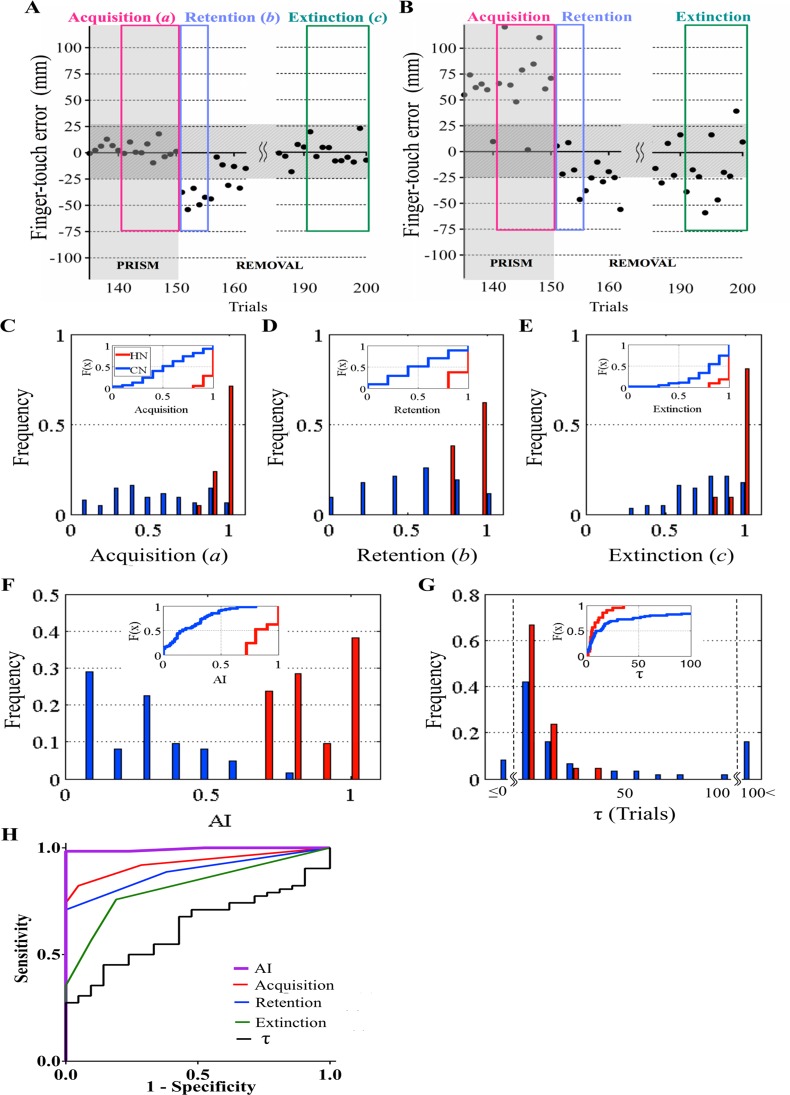
Quantitative evaluation of prism adaptation. (A) An example of adaptation in a healthy subject (HN13) shown in Fig. 2A. The finger-touch error of the last 10 trials of PRISM, and that of the initial five and last 10 trials of REMOVAL are extracted from Fig. 2A. Acquisition, retention, and extinction of adaptation were estimated from the probability of success (*a*) in the last 10 trials of PRISM (10/10), the probability of failure (*b*) in the initial five trials of REMOVAL (5/5), and the probability of success (*c*) in the last 10 trials of REMOVAL (10/10), respectively. *AI* was calculated as *a* × *b* × *c* and 1 in this case. (B) Similar analysis in CN4 shown in Fig. 2B. *a* = 1/10, *b* = 1/5, *c* = 6/10. *AI* = (1/10) × (1/5) × (6/10) = 0.012. Horizontally shaded areas in (A) and (B) represent the zone of “correct” touch (within ± 25mm). (C)–(F) Frequency distributions of *a* (C), *b* (D), *c* (E), and *AI* (F). Insets represent cumulative frequency curves. *F*(*x*) represents normal cumulative distribution function. (G) Frequency distribution of the time constant *τ* (for details, see Materials and Methods). Insets represent cumulative frequency curves of *τ*. Red columns and lines in (C)–(G) show data for 21 HN subjects. Blue columns and lines in (C)–(G) show data for 62 CN patients. (H) Receiver operating characteristic (ROC) curve analysis in the HN and CN groups. A purple line shows ROC curve for *AI*, a red line for the probability of acquisition, a blue line for the probability of retention, a green line for the probability of extinction, and a black line for *τ*.

We analyzed whether the following three probabilities [acquisition (“*a*”), retention (“*b*”), and extinction (“*c*”)] of adaptation could be quantitative markers for adaptation. Figs. 4A and 4B respectively show the three probabilities in a healthy subject (HN13 in Fig. 2A) and a SCA6 patient (CN4 in Fig. 2B). When we analyzed the HN and CN groups in terms of each of the three probabilities, the two groups showed statistically significant differences in all the three probabilities (inset of Figs. 4C-E, *p* < 0.0001, Kolmogorov-Smirnov test). However, a significant overlap was recognized at the range of 0.7–1 in their frequency distributions (Figs. 4C-E), suggesting that any of these three is insufficient to discriminate between normal and impaired motor learning. In contrast, the frequency distributions of *AI*, calculated as *a* × *b* × *c*, completely separated the HN and CN groups around the *AI* of 0.7 (Fig. 4F, *p* < 0.0001, Kolmogorov-Smirnov test), except for one patient with a pure parkinsonian MSA whose *AI* was 0.8 (CN59, Table 1). However, the patient was exceptional because the cerebellar dysfunction was not observed in this patient, and the magnetic resonance imaging (MRI) did not show any cerebellar or pontine atrophy.

We also calculated the time constant (*τ*) for the decay of the finger-touch error in PRISM for all the HN subjects and CN patients (Fig. 4G). Again, the frequency distributions of *τ* largely overlapped in the range of 1–30 trials between the HN and CN groups (median, 5.0 trials for HN and 11.3 trials for CN, *p* = 0.10, Mann-Whitney U-test), and thus no significant difference was recognized between these two groups in the cumulative frequency distribution (inset of Fig. 4G, *p* = 0.08, Kolmogorov-Smirnov test).

Analysis of the ROC curve further demonstrated a significantly high accuracy of discrimination between the HN and CN groups when using *AI* (AUC: 0.99). DeLong's test proved that *AI* powerfully discriminated the two groups compared with *a* (AUC: 0.93, *p* < 0.01), *b* (AUC: 0.89, *p* < 0.001), *c* (AUC: 0.82, *p* < 0.0001) and *τ* (AUC: 0.63, *p* < 0.0001) (Fig. 4H). Participants with *AI* < 0.68 belonged to the CN group with a sensitivity of 98.4% and a specificity of 100%, when the cutoff value determined from the ROC curve was applied as previously described [36]. These results led us to conclude that *AI* is the most reliable quantitative marker for the cerebellum-dependent motor learning in humans, and helps to discriminate the HN group from the CN group.

### Motor learning (*AI*) and incoordination

**Fig 5:**
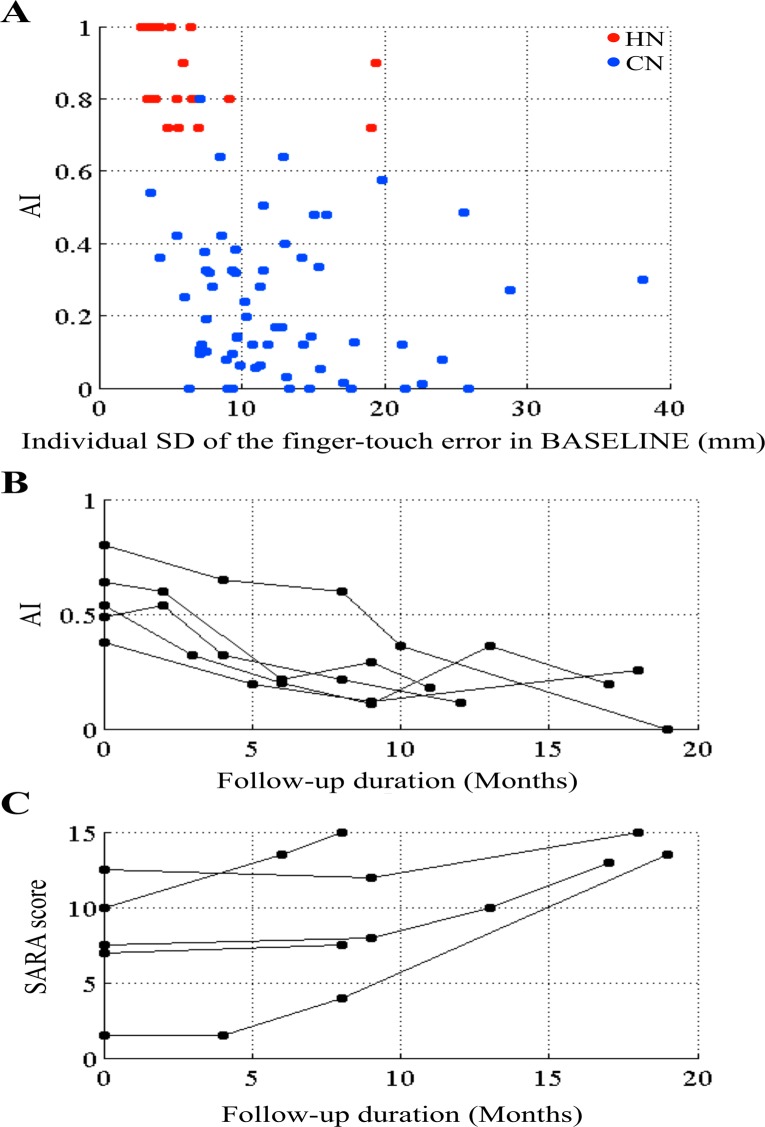
Relationship between *AI* and incoordination. (A) *AI* and the magnitude of dysmetria represented by the SD of the finger-touch error in BASELINE. Data were obtained from 62 CN patients (blue dots) and HN subjects (red dots). Each point represents data obtained from one subject. (B) and (C) Tracking *AI* (B) and SARA (C) data of each MSA patient (CN56, CN57, CN59, CN60, and CN61; n = 5).

The variability of the finger-touch error represents dysmetria, a sign of incoordination. We examined the relationship between *AI* and incoordination on the basis of the dysmetria represented by the variability of the finger-touch error in the 50 trials of BASELINE for individual subjects. As shown in Fig. 5A, *AI* appeared independent of the magnitude of dysmetria in the hand-reaching task in the HN or CN group, because no correlation was demonstrated between *AI* and SD of the hand-reaching error (HN, *r*_s_ = -0.30, *p* = 0.19; CN, *r*_s_ = -0.13, *p* = 0.33). However, when we tracked *AI* in five MSA patients (CN56, CN57, CN59, CN60, and CN61) for follow-up testing, *AI* decreased significantly within two years (*r*_s_ = -0.76, *p* < 0.0001, Fig. 5B). These patients were selected because disease progression is known to be much faster in MSA patients than in those with any other hereditary cerebellar ataxia [37,38]. By clinical inspection, ataxia apparently became exacerbated over time in these patients. Actually, the SARA score increased in all five patients during the follow-up period (Fig. 5C). Thus, *AI* did not correlate with the magnitude of incoordination for individual patients, but it correlated with the progression of incoordination when tracked longitudinally for each patient.

### *AI* distribution in healthy subjects and cerebellar patients

**Fig 6:**
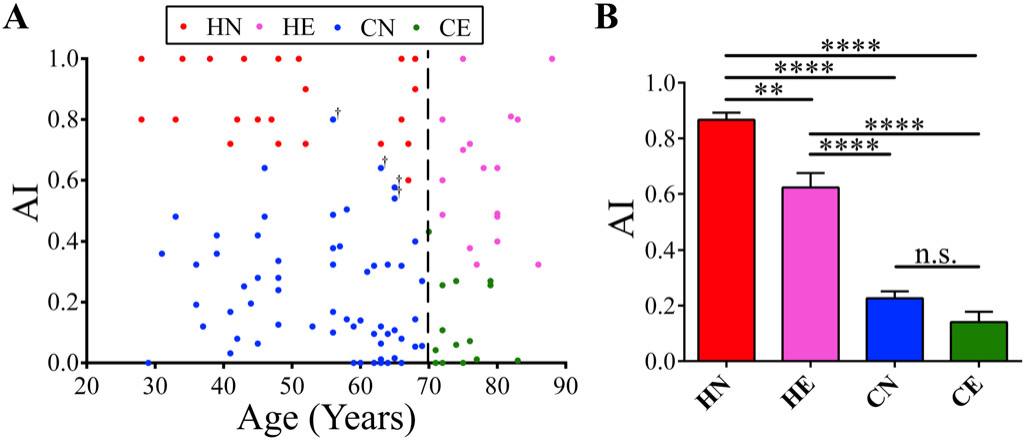
*AI* of healthy subjects (HN and HE) and cerebellar patients (CN and CE). (A) Distribution of *AI*s and ages for all the participants analyzed. *AI* tended to decrease and showed a widespread distribution in the HE group. Cerebellar patients (CN and CE) showed lower *AI*s than the age-matched healthy subjects (HN and HE). † indicates four pure parkinsonian MSA patients without clinical cerebellar signs. (B) Comparison of *AI* among the HN, HE, CN and CE groups. In all panels, red circles and columns represent HN; magenta, HE; blue, CN; and green, CE. **p < 0.01, ****p < 0.0001, Kruskal-Wallis test or Steel-Dwass test. Error bar represents SEM.

By comparing the *AI* of the healthy subjects among decade-wide age groups from 20s to 80s, we found a significant decrease in *AI* in the HE group (*p* < 0.05, Kruskal-Wallis test). *AI* in the HE group showed a wider distribution compared to those in the HN group (Fig. 6A, *p* < 0.05, Ansari-Bradley test). Moreover, as shown in Fig. 6B, clear differences in *AI* (*p* < 0.0001, Kruskal-Wallis test) were observed among healthy subjects (HN and HE) and patients with cerebellar diseases (CN and CE): *AI* of the HN group (0.867 ± 0.026, n = 21) was higher than those of the HE group (0.623 ± 0.052, n = 17; *p* < 0.01, Steel-Dwass test), CN (0.227 ± 0.024, n = 62; *p* < 0.0001, Steel-Dwass test), and CE (0.141 ± 0.037, n = 15; *p* < 0.0001, Steel-Dwass test) groups. There was no significant difference in *AI* between the CN and CE groups (*p* = 0.35, Steel-Dwass test).

### Comparison of *AI* with other clinical indices of cerebellar ataxia

**Fig 7:**
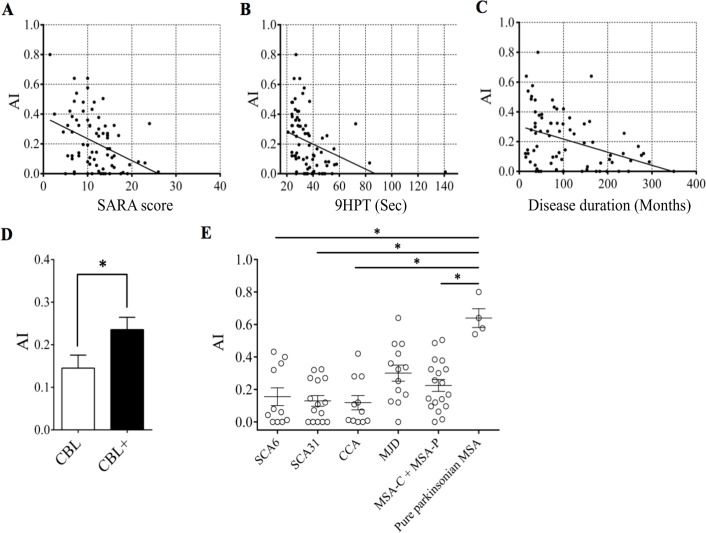
*AI* and other clinical indexes in various cerebellar diseases. (A)–(C) Scatter plots comparing *AI* with SARA score (A), 9-Hole Peg Test (B), and disease duration (C) in CN and CE patients. Linear regression lines are overlaid. (D) Comparison of *AI* between the CBL (n = 24) and CBL+ (n = 32) groups. *p < 0.05 by Mann-Whitney U-test. Error bar represents SEM. (E) *AI* was significantly higher in pure parkinsonian MSA patients than in SCA6, SCA31, CCA, or MSA (MSA-C and MSA-P) patients. *p < 0.05, post hoc Steel-Dwass test.

In the CN and CE groups, a negative correlation was observed between *AI* and SARA score (*r*_s_ = -0.34, *p* < 0.01, Fig. 7A), and between *AI* and 9HPT (*r*_s_ = -0.53, *p* < 0.0001, Fig. 7B). Consistent with the observation that degenerative diseases progress over time [37–40], a negative correlation was observed between *AI* and the duration of disease (*r*_s_ = -0.37, *p* < 0.001, Fig. 7C).

A comparison between the CBL and CBL+ groups showed that *AI* of the CBL group (0.145 ± 0.030) was smaller than that of the CBL+ group (0.236 ± 0.029, *p* < 0.05, Mann-Whitney U-test; Fig. 7D). Meanwhile, no significant difference in SARA score (11.6 ± 1.2 for CBL vs 11.2 ± 0.6 for CBL+, *p* = 0.98, Mann-Whitney U-test) or 9HPT (39.5 ± 4.9 s vs 34.4 ± 1.7 s, *p* = 0.71, Mann-Whitney U-test) was found between them. *AI* varied significantly among the pure cerebellar SCA patients with relatively mild ataxia showing SARA scores between 10 and 14 (S1 Fig.). These results suggest that *AI* is more sensitive for detecting changes in cerebellar functions than SARA score and 9HPT. Moreover, *AI* was significantly higher in pure parkinsonian MSA patients than in SCA6, SCA31, CCA, and MSA (MSA-C and MSA-P) patients (*p* < 0.05, Steel-Dwass test; Fig. 7E), which is consistent with this suggestion.

## Discussion

Taking advantage of the fact that the hand-reaching task is very simple, we succeeded in developing a paradigm for quantitatively assessing the cerebellum-dependent motor learning in almost any individual by performing 200 trials within only 20–30 minutes with a relatively cheap and compact system (S2 Table). A novel neurological biomarker (*AI*), which reflects acquisition, retention, and extinction of the prism adaptation of hand-reaching movement, was lower and its variability was larger in patients with cerebellar diseases than those in the age-matched healthy subjects. Moreover, *AI* decreased with aging even in healthy subjects over 70 years old. On the basis of these findings, we suggest *AI* as a new clinical index for the quantification of cerebellar motor learning.

### Characteristics of *AI*

The magnitude of motor errors at the initial portion of REMOVAL, the retention (*b*) in the present study, has been focused on for evaluating prism adaptation in previous studies [13, 41–43]. The present ROC analysis (Fig. 4H) proved that probabilities of the acquisition (*a*), retention (*b*) and extinction (*c*) of adaptation are all helpful for differentiating the healthy (HN) group from the ataxic (CN) group, but insufficient as a standalone single clinical maker for discriminating between the HN and CN groups. However, *AI* was a nearly perfect parameter with a cutoff value of 0.68 for the subjects below the age of 70 for detecting patients with cerebellar diseases. Taken together, we propose that *AI* is a reliable quantitative parameter of cerebellar function based on motor learning.

### Neural mechanisms involved in prism adaptation

Previous studies suggest that prism adaptation process can be divided into two phases: the early phase in which the subjects strategically, consciously and rapidly achieve error correction of hand movement within few trials in the early prism exposure, and the late phase in which they autonomously, unconsciously and slowly recalibrate spatial misalignments among distorted visual and incoming sensorimotor information, which requires a prolonged prism exposure [44, 45]. These two phases overlap during the process of prism adaptation. In PRISM of the present study, the early phase and the late phase may correspond to the decay of finger-touch error (*τ*) and the acquisition (*a*), respectively. The decay of finger-touch errors in the early phase (*τ*) was slow, and the acquisition (*a*) in the late phase was gradual and incomplete in patients with cerebellar diseases, implying that the cerebellum is involved in both phases. However, the acquisition (*a*) was more severely depressed than the decay of finger-touch errors (τ) (Figs. 2E, 2F, 4C and 4G). This implies that the contribution of the cerebellum may be larger in the late phase than in the early phase. It is assumed that the cerebellum is not the sole responsible brain area for prism adaptation. Several functional MRI (fMRI) and positron emission tomography (PET) studies [46–49], as well as a model study [50], have suggested that the cerebral cortex may be involved in the prism adaptation in addition to the cerebellum. Particularly, the fMRI studies consistently suggest that both the parietal cortex and cerebellar cortex are activated in the early phase of prism adaptation [47–49]. Taken together these previous studies, the result of the present study may suggest that both the cerebellum and parietal cortex contributes in the early phase of adaption, while only the cerebellum contributes in the late phase of adaptation. However, since most of the patients used in the present study have long history of movement disorders induced by cerebellar diseases, a possibility remains that some compensatory mechanisms, probably through the cerebral cortex, operated in prism adaptation. Hence, to determine the relative contributions of the cerebellum and parietal cortex in the early phase of prism adaptation, further studies of fMRI and patients of acute focal lesions are necessary.

The responsible areas of prism adaptation are not identified well in the cerebellum. Monkey lesion [51] and pharmacological reversible inactivation [17] studies consistently suggested that cerebellar hemispheric lobules VII (crus I and crus II), VIII (paramedian lobule and dorsal paraflocculus), vermal IX (uvula), and the dentate nucleus are involved in prism adaptation of the hand-reaching. A monkey unit-recoding study suggested that Purkinje cells in the cerebellar hemispheric lobules IV–VI encode hand-reaching error signals [52]. Clinical studies of cerebellar lesions suggest that the hemispheric lobules IV, V and VI are involved in adaptation of hand-reaching [53, 54]. A recent fMRI study has suggested that the hemispheric lobules III, IV, V, VI, VII, VIII and IX are activated in the early or late phases of the prism adaptation [55]. To determine the responsible cerebellar areas of prism adaptation, further studies of patients of acute focal cerebellar lesions are necessary.

### Relationship between motor learning and incoordination

Dysmetria is a symptom of incoordination induced by impaired cerebellar precision control. Studies of monkey saccade eye movements have shown that dysmetria, which is evaluated by the variability of movement in each trial, closely correlates with motor learning. Lesions of the monkey cerebellar vermal or hemispheric areas involved in saccade control not only impair the saccade amplitude adaptation but also increase the variability of saccade amplitude [56, 57]. In the present study, the variability of the finger-touch error induced by a prism decreased as the adaptation progressed in healthy subjects (Fig. 3A), indicating that motor learning may act to decrease the variability of movement. Meanwhile, patients with cerebellar diseases showed an increase in the variability of the finger-touch error in BASELINE compared with healthy subjects, indicating dysmetria. Such an increase in the variability of the finger-touch error in cerebellar patients did not improve during PRISM (Fig. 3B). While no correlation was found between *AI* and the magnitude of the variability of the finger-touch error for individual subjects (Fig. 5A), *AI* showed a tendency to decrease as the disease progressed in some patients (Figs. 5B and 5C). Taken together with these findings, we consider that there may be a close correlation between the impairment of motor learning and dysmetria. The possibility that limb ataxia may hamper cerebellar motor learning in cerebellar patients is unlikely, because very low *AI*s (0–0.2) were observed in individuals with a small variability of the finger-touch error (< 10 mm). Conceptually, the internal model of movement formed by motor learning is assumed to be utilized for precision motor control by the cerebellum [1, 58].

### Clinical implications of *AI*

The present prism adaptation paradigm provides several clinical implications. First, testing motor learning may help in extracting the cerebellar component from signs composed of multiple neural dysfunctions. In the present cohort, *AI* was lower in patients in the CBL group showing purely cerebellar syndromes than in the CBL+ group with clinical evidence of multisystem degenerations, whereas SARA and 9HPT did not show any significant difference between them (Fig. 7D). We reasoned that both SARA score and 9HPT reflect not only the cerebellar but also the extra-cerebellar signs such as bradykinesia of basal ganglia origin. This view is further supported by the comparison of *AI*, SARA and 9HPT between the MSA-C +MSA-P and pure parkinsonian MSA patients (Fig. 7E). Second, *AI* appears useful in quantitatively tracking the progression of changes in cerebellar dysfunctions even for two-year follow-up (Figs. 5B and 7C). To further confirm the utility of *AI* in tracking disease progression, much longer follow-up studies are necessary. Third, *AI* and the present paradigm may be applied to the rehabilitation of cerebellar dysfunctions, such as intensive coordination training with kinetic video games [59], which was shown to improve the motor performance of patients with cerebellar diseases.

*AI* was significantly decreased and dispersed in healthy subjects over 70 years old, suggesting that the aging effect on motor learning varied among individuals. The cerebellum is one of the brain regions that has been shown to decline with aging both anatomically [60, 61] and functionally [62–65]. A previous study of prism adaptation of ball throwing behavior showed that adaptation became slower [66, 67], which is generally consistent with the results of the present study.

The cerebellum has been suggested to be involved in cognitive functions such as emotional working memory [2], language [68–71], and thought [72]. The cooperation between the cerebellum and the cerebral cortex is assumed to be involved in such cognitive functions, as well as the induction of prism adaptation [47–50]. The present prism adaptation protocol and *AI* could thus be utilized not only to diagnose patients with cerebellar diseases, but also might help to investigate higher cerebellar functions based on the cerebro-cerebellar network loop.

## Supporting Information

S1 Fig Adaptation curves in mildly ataxic individuals. (A)-(D) The four panels show adaptation curves in mildly ataxic individuals [CN1 (A), CN2 (B), CN12 (C), and CN13 (D)], whose SARA scores are 10 to 14. (E) Summary of results.
(TIF)

S1 Table Characteristics of healthy subjects. The healthy participants included in this study consisted of 21 non-elderly (< 70 years old, HN) and 17 elderly (> 70, years old, HE) subjects. M = male; F = female; R = right; L = left; SARA = Scale for the Assessment and Rating of Ataxia; *AI* = adaptability index.
(DOC)

S2 Table Advantages of present paradigm in comparison with those used in previous studies. Fukushima K, Tanaka M, Suzuki Y, Fukushima J, Yoshida T. Adaptive changes in human smooth pursuit eye movement. Neurosci Res. 1996; 25: 391–398.
Shelhamer M, Tiliket C, Roberts D, Kramer PD, Zee DS. Short-term vestibulo-ocular reflex adaptation in humans. II. Error signals. Exp Brain Res. 1994; 100: 328–336.
Smith MA, Shadmehr R. Intact ability to learn internal models of arm dynamics in Huntington's disease but not cerebellar degeneration. J Neurophysiol. 2005; 93: 2809–2821.
Thach WT, Goodkin HP, Keating JG. The cerebellum and the adaptive coordination of movement. Annu Rev Neurosci. 1992; 15: 403–442.
Wallman J, Fuchs AF. Saccadic gain modification: visual error drives motor adaptation. J Neurophysiol. 1998; 80: 2405–2416.
Woodruff-Pak DS, Papka M, Ivry RB. Cerebellar involvement in eyeblink classical conditioning in humans. Neuropsychology. 1996; 10: 443–458.
(DOC)
