# Supplementary figures and images for: Quantitative Evaluation of Human Cerebellum-Dependent Motor Learning through Prism Adaptation of Hand-Reaching Movement
Source: PLoS One. 2015 Mar 18;10(3):e0119376. doi: 10.1371/journal.pone.0119376 (PMC4364988; doi:10.1371/journal.pone.0119376)

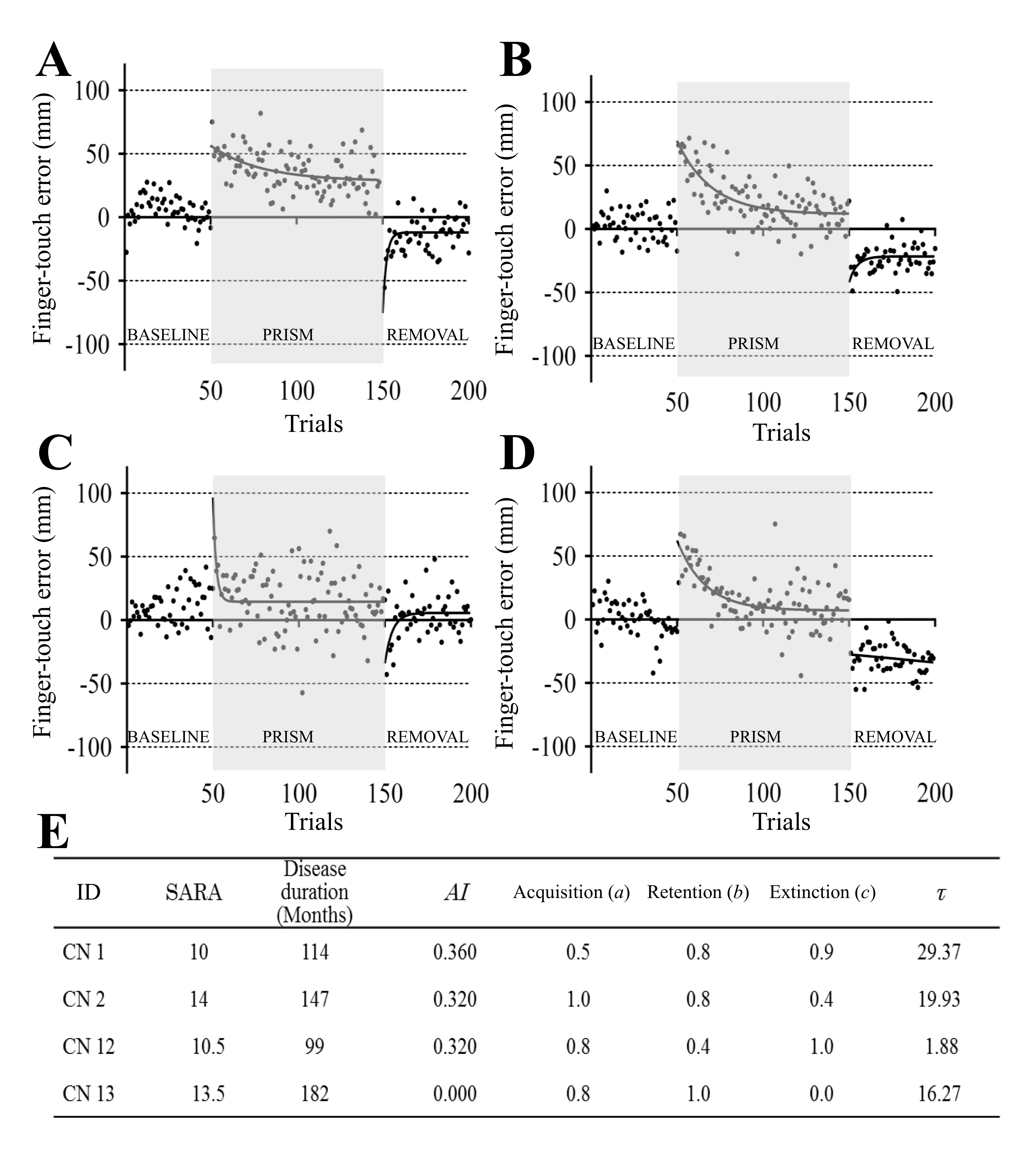

Supplement: S1 Fig — (A)-(D) The four panels show adaptation curves in mildly ataxic individuals [CN1 (A), CN2 (B), CN12 (C), and CN13 (D)], whose SARA scores are 10 to 14. (E) Summary of results. (TIF) [file pone.0119376.s001.tif]
