# Supplementary material for: Quantitative Evaluation of Human Cerebellum-Dependent Motor Learning through Prism Adaptation of Hand-Reaching Movement
Source: PLoS One. 2015 Mar 18;10(3):e0119376. doi: 10.1371/journal.pone.0119376 (PMC4364988; doi:10.1371/journal.pone.0119376)
Supplement: S1 Table — The healthy participants included in this study consisted of 21 non-elderly (< 70 years old, HN) and 17 elderly (> 70, years old, HE) subjects. M = male; F = female; R = right; L = left; SARA = Scale for the Assessment and Rating of Ataxia; AI = adaptability index. (DOC) [file pone.0119376.s002.doc]

| **Healthy Non-Elderly (HN)** | | | | **Healthy-Elderly (HE)** | | | |
| --- | --- | --- | --- | --- | --- | --- | --- |
| ID | Age (year) / Gender / Handedness | SARA | *AI* | ID | Age (year) / Gender / Handedness | SARA | *AI* |
| HN 1 | 28 / F / R | 0 | 0.800 | HE1 | 72 / F / L | 0 | 0.800 |
| HN 2 | 28 / F / R | 0 | 1.000 | HE2 | 72 / M / R | 0 | 0.486 |
| HN 3 | 33 / M / R | 0 | 0.800 | HE3 | 72 / F / R | 0 | 0.600 |
| HN 4 | 34 / M / R | 0 | 1.000 | HE4 | 75 / M / R | 0 | 0.700 |
| HN 5 | 38 / M / R | 0 | 1.000 | HE5 | 75 / M / R | 0.5 | 1.000 |
| HN 6 | 41 / F / R | 0 | 0.720 | HE6 | 76 / F / R | 0 | 0.378 |
| HN 7 | 42 / F / R | 0 | 0.800 | HE7 | 76 / F / R | 0.5 | 0.720 |
| HN 8 | 43 / M / R | 0 | 1.000 | HE8 | 77 / F / R | 1 | 0.324 |
| HN 9 | 45 / F / R | 0 | 0.800 | HE9 | 78 / M / R | 0 | 0.640 |
| HN 10 | 47 / M / R | 0 | 0.800 | HE10 | 80 / M / R | 1 | 0.480 |
| HN 11 | 48 / F / R | 0 | 0.720 | HE11 | 80 / F / R | 2 | 0.490 |
| HN 12 | 48 / M / R | 0 | 1.000 | HE12 | 80 / M / R | 1 | 0.640 |
| HN 13 | 51 / F / R | 0 | 1.000 | HE13 | 80 / F / R | 0.5 | 0.400 |
| HN 14 | 52 / F / R | 0 | 0.900 | HE14 | 82 / M / R | 1 | 0.810 |
| HN 15 | 52 / F / R | 0 | 0.720 | HE15 | 83 / F / R | 1.5 | 0.800 |
| HN 16 | 63 / F / R | 0 | 0.720 | HE16 | 86 / F / R | 0.5 | 0.324 |
| HN 17 | 66 / F / R | 0 | 0.800 | HE17 | 88 / M / L | 3 | 1.000 |
| HN 18 | 66 / M / R | 0 | 1.000 |  |  |  |  |
| HN 19 | 67 / F / R | 0 | 0.720 |  |  |  |  |
| HN 20 | 68 / M / L | 0 | 1.000 |  |  |  |  |
| HN 21 | 68 / M / R | 0 | 0.900 |  |  |  |  |
