# Supplementary material for: Quantitative Evaluation of Human Cerebellum-Dependent Motor Learning through Prism Adaptation of Hand-Reaching Movement
Source: PLoS One. 2015 Mar 18;10(3):e0119376. doi: 10.1371/journal.pone.0119376 (PMC4364988; doi:10.1371/journal.pone.0119376)
Supplement: S2 Table — Fukushima K, Tanaka M, Suzuki Y, Fukushima J, Yoshida T. Adaptive changes in human smooth pursuit eye movement. Neurosci Res. 1996; 25: 391–398. Shelhamer M, Tiliket C, Roberts D, Kramer PD, Zee DS. Short-term vestibulo-ocular reflex adaptation in humans. II. Error signals. Exp Brain Res. 1994; 100: 328–336. Smith MA, Shadmehr R. Intact ability to learn internal models of arm dynamics in Huntington's disease but not cerebellar degeneration. J Neurophysiol. 2005; 93: 2809–2821. Thach WT, Goodkin HP, Keating JG. The cerebellum and the adaptive coordination of movement. Annu Rev Neurosci. 1992; 15: 403–442. Wallman J, Fuchs AF. Saccadic gain modification: visual error drives motor adaptation. J Neurophysiol. 1998; 80: 2405–2416. Woodruff-Pak DS, Papka M, Ivry RB. Cerebellar involvement in eyeblink classical conditioning in humans. Neuropsychology. 1996; 10: 443–458. (DOC) [file pone.0119376.s003.doc]

|  | **Previous paradigms** | **Present paradigm** |
| --- | --- | --- |
| **Paradigm** | **1** **VOR (vestibulo-ocular reflex)** (Shelhamer et al., 1994) | **Prism adaptation (hand-reaching)** |
|  | **2 Eyeblink conditioning** (Woodruff-Pak et al., 1996) |  |
|  | **3 Smooth pursuit eye movement** (Fukushima et al., 1996) |  |
|  | **4 Saccadic eye movement** (Wallman and Fuchs, 1998) |  |
|  | **5 Prism adaptation (dart throwing)** (Thach et al., 1992) |  |
|  | **6 Force field adaptation** (Smith and Shadmehr, 2005) |  |
| **Space needed** | **Large (3 × 3 m^2^)** | **Small (1 × 1 m^2^)** |
| **Set-up cost** | **Expensive (maximally $50,000)** | **Cheap ($10,000)** |
| **Time required for data sampling** | **Long (more than 1 hour)** (Shelhamer et al., 1994) |  |
|  | **Short** (Woodruff-Pak et al., 1996, Thach et al., 1992, Fukushima et al., 1996, Wallman and Fuchs, 1998, Smith and Shadmehr, 2005) | **Short (20–30 min)** |
| **Time required for data analysis** | **Off-line** | **Real time (on-line)** |
| **Task characteristics** | **Reflex** (Shelhamer et al., 1994, Woodruff-Pak et al., 1996) |  |
|  | **Voluntary movement** (Thach et al., 1992, Fukushima et al., 1996, Wallman and Fuchs, 1998, Smith and Shadmehr, 2005) | **Voluntary movement** |
|  | **Easy for everyone** (Shelhamer et al., 1994, Fukushima et al., 1996, Woodruff-Pak et al., 1996, Wallman and Fuchs, 1998) | **Simple and feasible** |
|  | **Not easy** (Thach et al., 1992, Smith and Shadmehr, 2005) |  |
|  | **Invasive** (Woodruff-Pak et al., 1996) |  |
|  | **Non-invasive** (Thach *et al.*, 1992, Shelhamer *et al.*, 1994, Fukushima *et al.*, 1996, Wallman and Fuchs, 1998, Smith and Shadmehr, 2005) | **Non-invasive** |
